# Exploration of the novel fluoroquinolones with high inhibitory effect against quinolone-resistant DNA gyrase of *Salmonella* Typhimurium

**DOI:** 10.1128/spectrum.01330-23

**Published:** 2023-10-05

**Authors:** Jirachaya Toyting, Nami Miura, Fuangfa Utrarachkij, Wimonrat Tanomsridachchai, Lawrence P. Belotindos, Pondpan Suwanthada, Thoko Flav Kapalamula, Siriporn Kongsoi, Kentaro Koide, Hyun Kim, Jeewan Thapa, Chie Nakajima, Yasuhiko Suzuki

**Affiliations:** 1 Division of Bioresources, Hokkaido University International Institute for Zoonosis Control, Sapporo, Japan; 2 Department of Microbiology, Faculty of Public Health, Mahidol University, Bangkok, Thailand; 3 Biosafety and Environment Section, Research and Development Division, Philippine Carabao Center National Headquarters and Gene Pool Science City of Munoz, Munoz, Nueva Ecija, Philippines; 4 Department of Veterinary Public Health, Faculty of Veterinary Medicine, Kasetsart University, Nakhon Pathom, Thailand; 5 Department of Bacteriology II, National Institute of Infectious Diseases, Tokyo, Japan; 6 Hokkaido University Institute for Vaccine Research & Development, Hokkaido University, Sapporo, Japan; 7 International Collaboration Unit, Hokkaido University, International Institute for Zoonosis Control, Sapporo, Japan; AP-HP, Sorbonne Université, Paris, France

**Keywords:** fluoroquinolones, DNA gyrase, *Salmonella *Typhimurium

## Abstract

Quinolone-resistant nontyphoidal *Salmonella*, one of the prominent pathogens causing acute gastroenteritis, has become a public health concern globally. The World Health Organization has ranked fluoroquinolone-resistant *Salmonella* as a high-priority pathogen for researching and developing new antibiotics. WQ-3034 and WQ-3154 are relatively new synthetic fluoroquinolones with distinctive structures. WQ-3034 has 6-amino-3,5-difluoropyridine-2-yl at R_1_, 3-hydroxyazetidinyl at R_7_, and the addition of chlorine atom at R_8_. WQ-3154 has a similar basic pharmacophore to WQ-3034 except for the modification at R_8_ with a methyl group. In this study, the inhibitory effect and DNA cleavage effect against wild-type (WT) and mutant *Salmonella* Typhimurium DNA gyrases of WQ-3034 and WQ-3154 were examined along with WQ-3810 and ciprofloxacin by measuring the drug concentration that inhibits half of the enzyme activity (IC_50_) and the drug concentration that induces 25% of maximum DNA cleavage (CC_25_). The minimum inhibitory concentration (MIC) of the compounds was assessed against *Salmonella* Typhimurium and *Salmonella* Enteritidis. Among four compounds, WQ-3034 demonstrated the highest inhibitory effect against both WT and mutant *Salmonella* Typhimurium DNA gyrases with amino acid substitution at codon 83 and/or 87, while ciprofloxacin showed the lowest inhibitory effect. Remarkably, WQ-3034 and WQ-3154 exhibited a significantly higher inhibitory effect than ciprofloxacin against *Salmonella* Typhimurium DNA gyrase with double amino acid substitution, Ser83Phe-Asp87Asn. Similarly, CC_25_ of WQ-3034 against mutant *Salmonella* Typhimurium DNA gyrase was lower than ciprofloxacin. Notably, MICs of WQ-3034 and WQ-3154 were higher than ciprofloxacin. In conclusion, this study revealed that WQ-3034 and WQ-3154 could potentially be effective therapeutic agents against quinolone-resistant nontyphoidal *Salmonella*.

**IMPORTANCE:**

Quinolone-resistant nontyphoidal *Salmonella* is a pressing public health concern, demanding the exploration of novel treatments. In this study, we focused on two innovative synthetic fluoroquinolones, WQ-3034 and WQ-3154. Our findings revealed that these new compounds demonstrate potent inhibitory effects, even against mutant strains that cause resistance to existing quinolones. Hence, WQ-3034 and WQ-3154 could potentially be effective therapeutic agents against quinolone-resistant *Salmonella* Typhimurium. Furthermore, the data obtained in this study will be baseline information for antimicrobial drug development.

## INTRODUCTION

Nontyphoidal *Salmonella* is one of the leading causative agents of acute gastroenteritis. In 2017, the Global Burden of Diseases, Injuries, and Risk Factors Study 2017 estimated that nontyphoidal *Salmonella* caused 95.1 million cases of gastroenteritis with 50,771 deaths worldwide (1, 2). Among more than 2,500 serotypes of *S. enterica, S.* Typhimurium and *S.* Enteritidis are the most common serotypes associated with foodborne illness (3, 4). Fluoroquinolones (FQs) are one of the drugs of choice for *Salmonella* infection. Nevertheless, the antimicrobial resistance rate of nontyphoidal *Salmonella* has been increasing dramatically over the past decade, particularly in *S.* Typhimurium (5, 6). Hence, antimicrobial-resistant nontyphoidal *Salmonella* has become a critical public health concern globally.

Quinolones are a class of synthetic antimicrobial agents containing a bicyclic core structure (7), which is used for the treatment of bacterial infection. To improve pharmacokinetics and extend their spectrum, various modifications have been processed to the quinolone core structure. FQs have been designated by a key modification, the addition of a fluorine atom at the R_6_ position of the bicyclic core structure (8). One of the most active FQs is ciprofloxacin, which has the addition of a cyclopropyl group to the R_1_ position and a piperazine ring to the R_7_ position to enhance its activity (8 – 10). However, the quinolone-resistant *Salmonella* is rising and being reported in various regions of the world, contributing to the high global burden of *Salmonella* infections (11). As such, the World Health Organization (WHO) precisely ranked fluoroquinolone-resistant *Salmonella* as a high-priority pathogen for the research and development of new antibiotics since 2017 (12).

**Fig 1:**
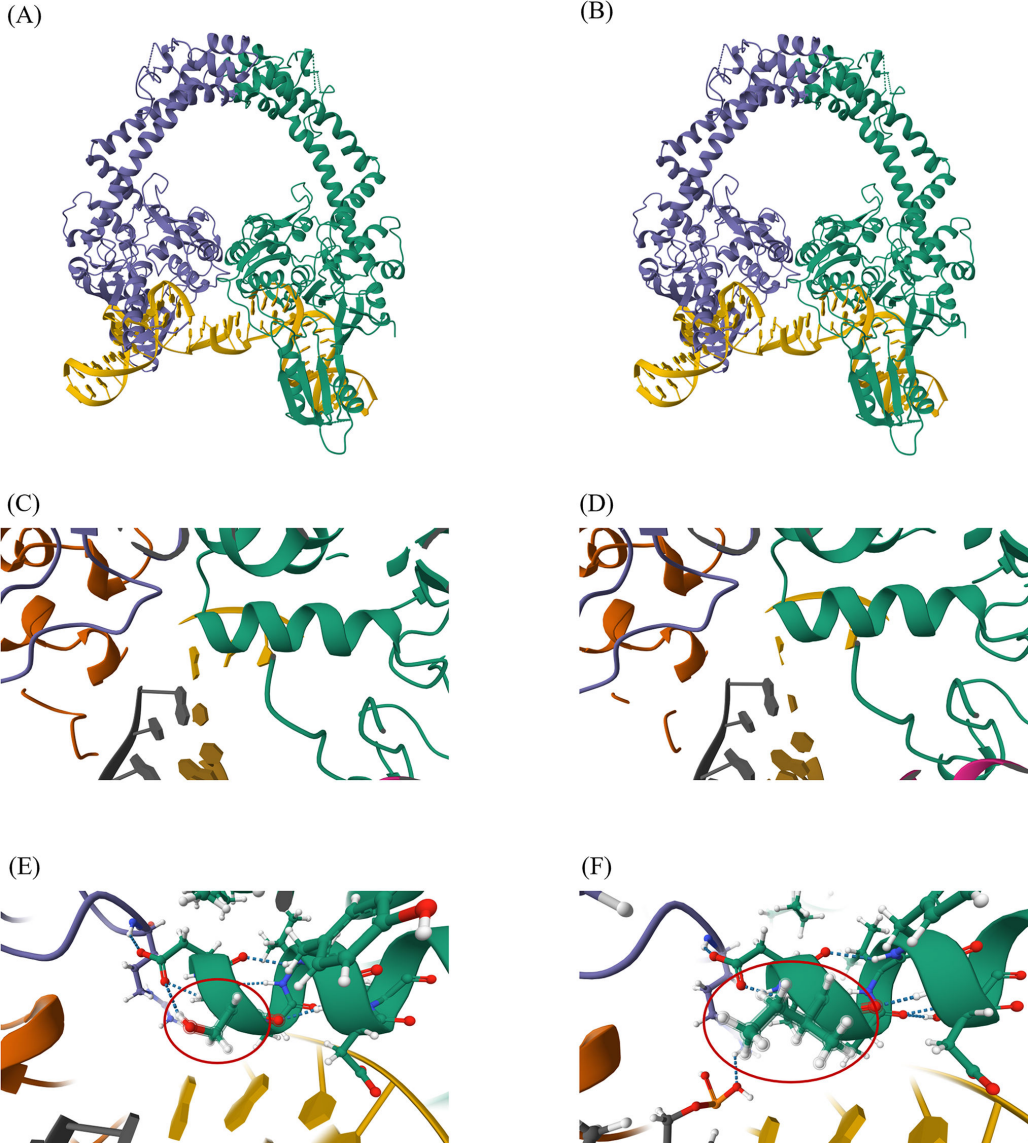
3D structures of *Salmonella* Typhimurium DNA gyrases, created using homology-based methods from *Escherichia coli*. The figure highlights the DNA gyrase A subunits (A, B), the structure of its alpha helix (C, D), and the structure of codon 83 in both WT and mutant Ser83Ile (E, F). The WT structures are represented in panels A, C, and E, while the mutant structures are shown in panels B, D, and F. The purple and green ribbons represent DNA gyrase A subunits. The double helix structure of DNA is shown in yellow; the white, red, and blue balls represent hydrogen, oxygen, and nitrogen atoms, respectively.

Quinolones act as antibacterial agents by disturbing bacterial DNA synthesis and inhibiting the replication pathway (13, 14). The intracellular targets of quinolones are two type II DNA topoisomerases, namely, DNA gyrase and DNA topoisomerase IV. DNA gyrase encompasses two pairs of GyrA/GyrB subunits that take part in bacterial DNA synthesis (15). Quinolones bind to DNA-DNA gyrase complex in a noncovalent manner at the cleavage-ligation active site and act as a physical block to prevent the ligation of bacterial DNA, resulting in replication process inhibition, DNA fragmentation, and cell death. The interactions between quinolones and DNA gyrase are mediated by a water-metal ion bridge, which is formed through essential contacts contributed by the carbonyl substituents at R_3_ and R_4_. This bridge comprises a noncatalytic Mg^2+^ ion coordinated with four water molecules and carbonyl oxygens at R_3_ and R_4_ of the quinolones, forming an octahedral complex. Two water molecules interact with enzyme residues Ser83 and Asp87 in GyrA, thus completing the bridge structure (10, 16). Nonetheless, the emergence of quinolones resistance has limited the use of these antibiotics. Mutation as amino acid substitution in the residues that anchor the bridge is the common mechanism of quinolones resistance. Amino acid substitutions in Ser83 and Asp87 of GyrA subunit, including Ser83Phe, Ser83Tyr, Asp87Gly, Asp87Asn, and Asp87Tyr, are the most common mutations associated with quinolone resistance in *Salmonella*. In addition, Ser83Ile and Ser87Phe-Asp87Asn confer high-resistance levels against FQs were also reported (17 – 23). Fig. 1 illustrates the contrasting structural models of the wild-type (WT) and mutated DNA gyrase. Hence, the development of novel antimicrobial agents showing a high affinity for mutated DNA gyrases is desirable.

**Fig 2:**
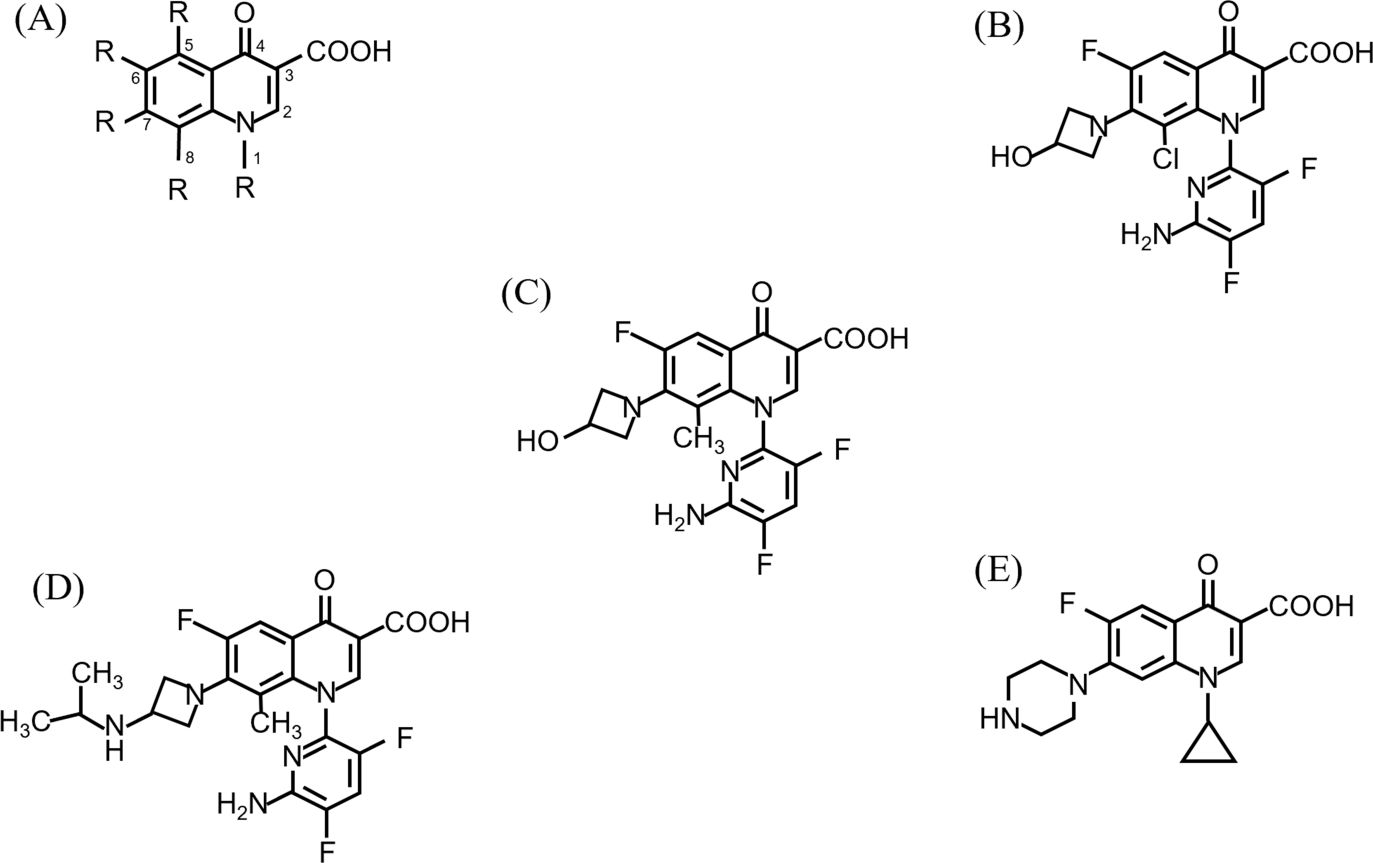
Chemical structures of the quinolones tested in this study. (A) Basic structure, (B) WQ-3034, (C) WQ-3154, (D) WQ-3810, and (E) ciprofloxacin.

WQ-3034 (also known as delafloxacin, ABT-492, RX-3341) is a relatively new synthetic fluoroquinolones, which received US Food and Drug Administration approval for Acute Bacterial Skin and Skin Structure Infections (ABSSSIs) therapy in 2017 (24). WQ-3034 has distinct structures including 6-amino-3,5-difluoropyridine-2-yl at the R_1_ position, 3-hydroxyazetidinyl at the R_7_ position, and chlorine (Cl) atom at the R_8_ position (Table 1; Fig. 2). The previous study revealed that WQ-3034 was the most potent compound tested against methicillin-susceptible and methicillin-resistant *Staphylococcus aureus*, *Streptococcus pneumoniae*, viridans group streptococci, and beta-hemolytic streptococci (25). WQ-3034 also showed more potency against quinolone-susceptible and quinolone-resistant gram-positive bacteria, while having similar activity to ciprofloxacin in certain *Enterobacteriaceae* members (26). In contrast to other quinolones, WQ-3034 is considered a double-targeting drug that can bind to DNA gyrase and DNA topoisomerase IV and displays equal affinity to both enzymes (27). With this property, WQ-3034 is expected to have good *in vitro* activity against both gram-positive and gram-negative bacteria. WQ-3810 possesses 3-isopropylaminoazetizine-1-yl at the R_7_ position and methyl group at the R_8_ position (Table 1; Fig. 2). Previous studies revealed potent antimicrobial activity of WQ-3810 against *Acinetobacter baumannii*, *Escherichia coli*, *Streptococcus pneumonia*, methicillin-resistant *Staphylococcus aureus*, and *Neisseria gonorrhoeae* (28). In addition, WQ-3810 showed a high inhibitory effect against DNA gyrase of *S.* Typhimurium (29) and *Mycobacterium leprae* (30). WQ-3154 has a similar basic pharmacophore to WQ-3034 except for the methyl group instead of Cl atom at the R_8_ position (Table 1; Fig. 2). Studies on WQ-3034 and *Salmonella* in terms of DNA gyrase inhibition activity and antimicrobial effect are very limited, particularly in mutant *Salmonella* DNA gyrases. In addition, DNA gyrase inhibitory activity and potency of WQ-3154 have never been evaluated before. Therefore, the objectives of this study were to assess and compare the inhibitory effect of WQ-3034 and WQ-3154 along with WQ-3810 and ciprofloxacin on wild-type and mutant *S.* Typhimurium DNA gyrases and further to understand the antimicrobial activity of these drugs against nontyphoidal *Salmonella*.

**TABLE 1:** Chemical structures of the quinolones tested in this study

Fluoroquinolones	R_1_	R_6_	R_7_	R_8_
WQ-3034	6-Amino-3,5-difluoropyridine-2-yl	F	3-Hydroxyazetidinyl	Cl
WQ-3154	6-Amino-3,5-difluoropyridine-2-yl	F	3-Hydroxyazetidinyl	CH_3_
WQ-3810	6-Amino-3,5-difluoropyridine-2-yl	F	3-Isopropylaminoazetizine-1-yl	CH_3_
CIP	Cyclopropyl	F	Piperazine	H

## RESULTS

### Inhibitory effect of FQs on *S.* Typhimurium recombinant DNA gyrase

IC_50_s estimated from fluoroquinolone-inhibited DNA gyrase supercoiling assay are summarized in Table 2, and gel electrophoresis patterns are presented in Fig. 4. IC_50_s of WQ-3034 against WT and all mutant *S*. Typhimurium DNA gyrase were found to be lowest, conversely IC_50_s of ciprofloxacin were found to be highest. WQ-3810 had lower IC_50_s than WQ-3145 against DNA gyrase with amino acid substitution at codon 87, on the other hand, WQ-3154 had lower IC_50_s than WQ-3810 at DNA gyrase with amino acid substitution at codon 83 and double mutation. Among single-mutant DNA gyrases, IC_50_s of all four FQs against Ser83Ile were the highest. IC_50_s of every fluoroquinolone against double-mutant DNA gyrase were significantly higher than WT and single-mutant DNA gyrase. For example, IC_50_s of ciprofloxacin against double-mutant *S*. Typhimurium DNA gyrase were 7,264-fold and 95-fold to 1,144-fold greater than that of WT and single-mutant DNA gyrase, respectively. Compared to ciprofloxacin, IC_50_s of WQ-3034, WQ-3154, and WQ-3810 against double-mutant DNA gyrases were comparatively lower. IC_50_s of WQ-3034, WQ-3154, and WQ-3810 against double-mutant DNA gyrase were 59-fold, 70-fold, and 110-fold greater than WT, respectively. However, these were considerably less than (1/50th) that of ciprofloxacin.

**TABLE 2:** IC_50_s of the quinolones against WT and mutant *Salmonella* DNA gyrases

Quinolones	IC_50_s ± SD (μg/mL)^*a*^ ^,^ ^*c*^							
	WT^*b*^	Ser83Ile	Ser83Phe	Ser83Tyr	Asp87Gly	Ap87Asn	Asp87Tyr	Ser83Phe-Asp87Asn
WQ-3034	0.020 ± 0.003	0.840 ± 0.029	0.026 ± 0.003	0.218 ± 0.023	0.024 ± 0.002	0.450 ± 0.066	0.030 ± 0.002	1.183 ± 0.132
WQ-3154	0.026 ± 0.005	1.154 ± 0.064	0.030 ± 0.002	0.249 ± 0.036	0.035 ± 0.003	0.875 ± 0.031	0.034 ± 0.002	1.813 ± 0.033
WQ-3810	0.033 ± 0.010	1.158 ± 0.223	0.052 ± 0.014	0.414 ± 0.083	0.032 ± 0.002	0.593 ± 0.121	0.033 ± 0.005	3.649 ± 0.468
Ciprofloxacin	0.072 ± 0.008	5.484 ± 0.182	0.457 ± 0.106	1.219 ± 0.012	0.631 ± 0.064	1.673 ± 0.145	0.668 ± 0.139	523.0 ± 161.1

^*a*^ IC_50_, concentration of quinolones that inhibit DNA gyrase activity by 50%.

^*b*^ WT, wild type.

^*c*^ SD, standard deviation.

**Fig 3:**
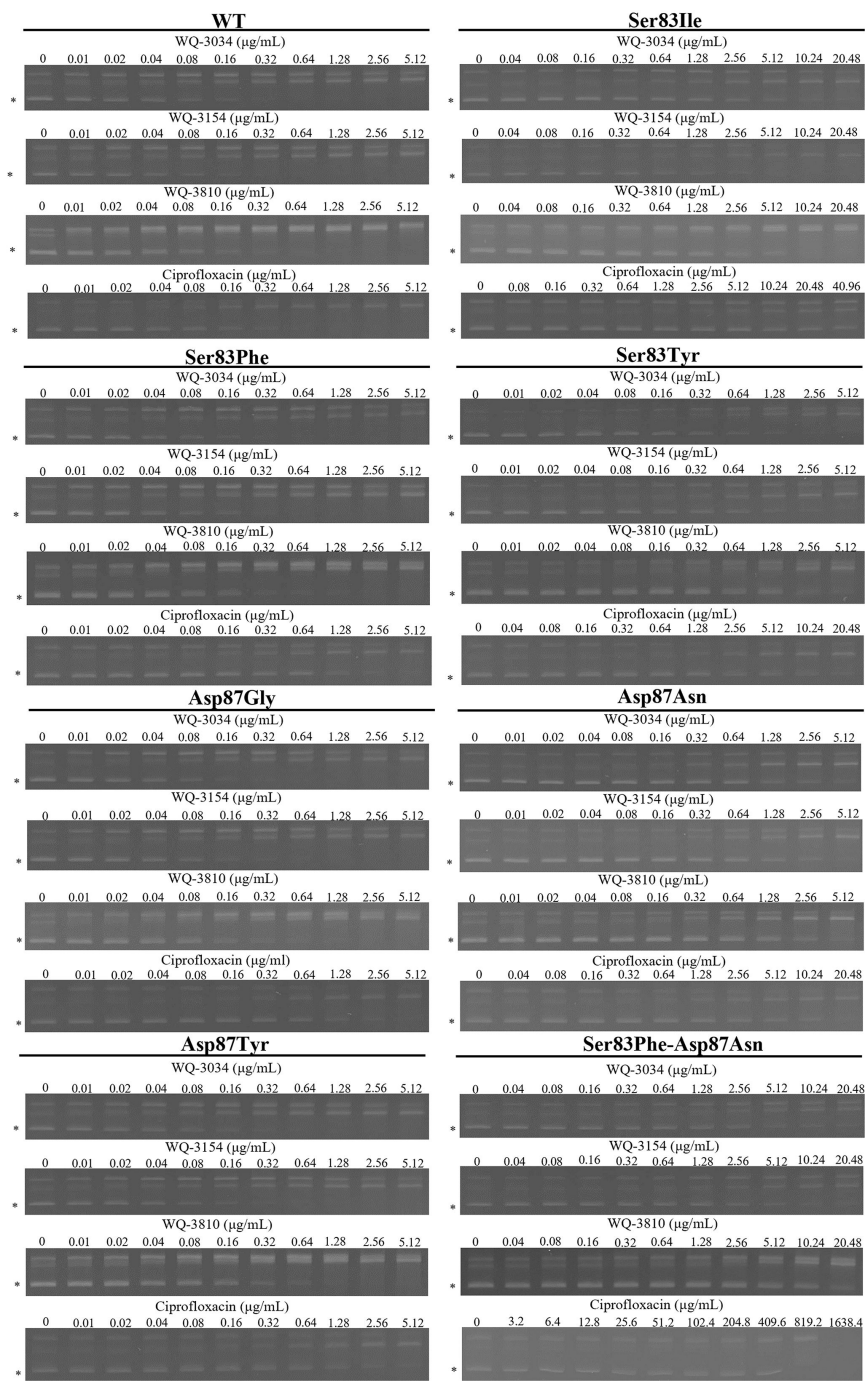
Inhibitory activity of WQ-3034, WQ-3154, WQ-3810, and ciprofloxacin. Asterisks (*) indicate the supercoiled DNA bands. Of note, as the concentration of quinolone increased, the intensity of the supercoiled DNA band decreased.

**Fig 4:**
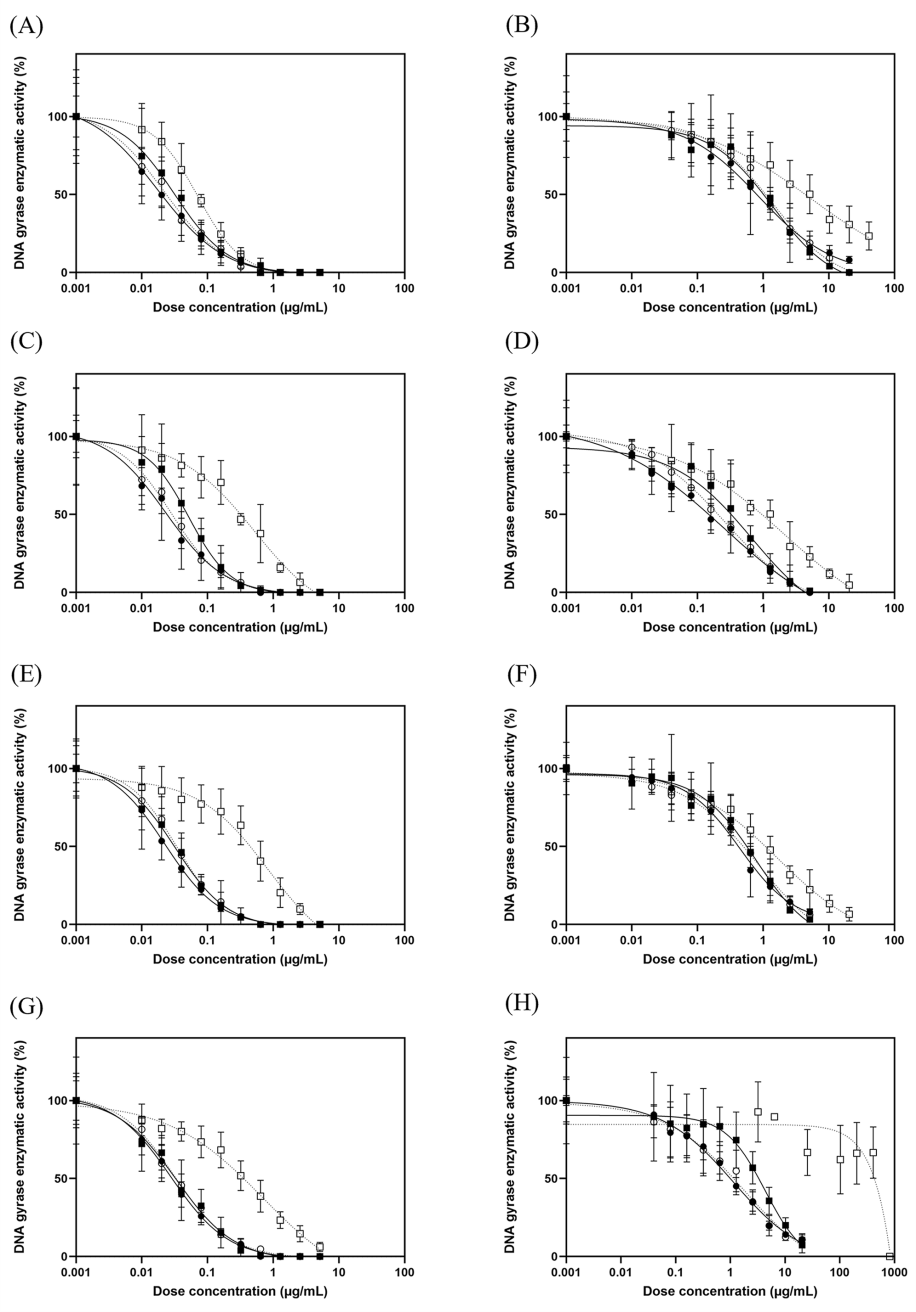
Sigmoidal graphs represent the inhibitory effect of fluoroquinolones against WT and mutant *S.* Typhimurium DNA gyrase in a dose-dependent manner. The inhibitory activity of WQ-3034, WQ-3154, WQ-3810, and ciprofloxacin against (A) WT, (B) Ser83Ile, (C) Ser83Phe, (D) Ser83Tyr, (E) Asp87Gly, (F) Asp87Asn, (G) Asp87Tyr, and (H) Ser83Phe-Asp87Asn. The solid circle, open circle, solid square, and open square denote WQ-3034, WQ-3154, WQ-3810, and ciprofloxacin, respectively.

As indicated in Fig. 3, all four fluoroquinolones exhibited the DNA gyrase inhibitory effect in a dose-dependent manner. The inhibitory activity trends of each fluoroquinolone against WT and seven mutant DNA gyrases are summarized in Fig. 4. The slope showed that when the fluoroquinolone concentration increased, the DNA gyrase enzymatic activity decreased. Generally, inhibitory activity trends were similar among WQ compounds against WT and mutant *S*. Typhimurium DNA gyrase except in double mutation, where WQ-3810 presented a different shape of the slope. In addition, ciprofloxacin also showed a distinct slope shape compared to WQ compounds.

### Fluoroquinolone-mediated DNA cleavage complex by WT and GryA-Ser83Ile DNA gyrases

**Fig 5:**
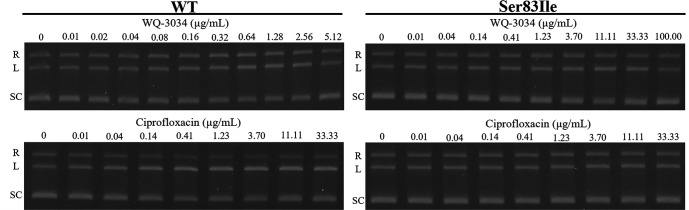
DNA cleavage activity of WQ-3034 and ciprofloxacin against WT and GryA-Ser83Ile *S.* Typhimurium DNA gyrases. R, L, and SC denote relaxed, linear, and supercoiled pBR322 DNA, respectively. Of note, as the concentration of quinolones increased, the intensity of linear DNA increased and then decreased after reaching the maximum DNA cleavage.

**Fig 6:**
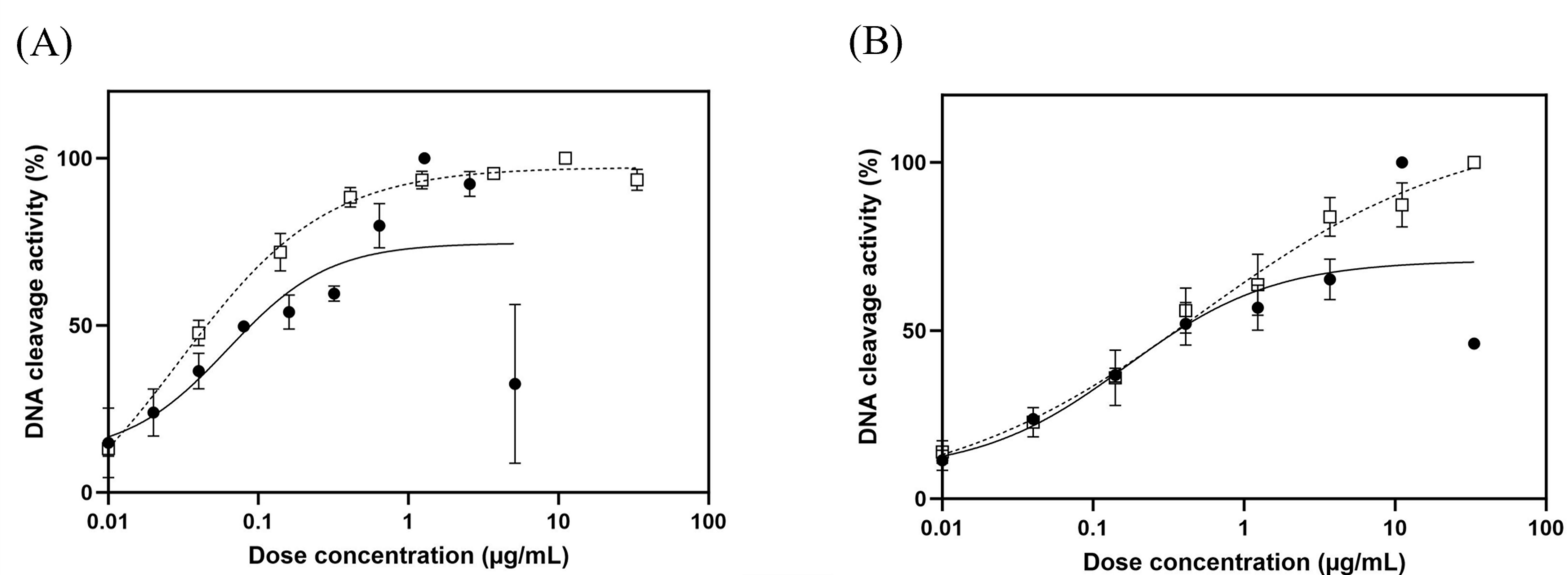
Graphs represent the DNA cleavage levels of fluoroquinolones against WT and mutant *S.* Typhimurium DNA gyrase. The DNA cleavage levels of WQ-3034 and ciprofloxacin against (A) WT and (B) Ser83Ile. The solid circle and open square denote WQ-3034 and ciprofloxacin, respectively.

Table 3 provides a summary of the CC_25_s obtained from the fluoroquinolone-mediated DNA cleavage assays. The gel electrophoresis patterns depicting these results are presented in Fig. 5, and the levels of DNA cleavage are summarized in Fig. 6. CC_25_s of WQ-3034 against the WT were found to be comparable to those of ciprofloxacin. However, when estimating the CC_25_ against Ser83Ile mutant DNA gyrase of WQ-3034, it was not possible to calculate the CC_25_ of ciprofloxacin for the same mutant. This was due to ciprofloxacin failing to induce maximum (100%) DNA cleavage at the equivalent concentration of WQ-3034. Thus, this could be inferred that WQ-3034 has a better DNA cleavage effect against Ser83Ile mutant DNA gyrase than ciprofloxacin.

**TABLE 3:** CC_25_s of the quinolones against WT and mutant *Salmonella* DNA gyrases^*a*^

Quinolones	CC_25_s (μg/mL)	
	WT^*b*^	Ser83Ile
WQ-3034	0.16	0.58
Ciprofloxacin	15	1.11

^*a*^ CC_25_, concentration of quinolones that induce 25% of maximum DNA cleavage.

^*b*^ WT, wild type.

### MICs of FQs against nontyphoidal *Salmonella*

Minimum inhibitory concentration (MICs) of four FQs are summarized in Table 4. WQ-3034 had slightly different MICs between against *S*. Typhimurium and *S*. Enteritidis at 0.08 and 0.16 µg/mL, while WQ-3154 had similar MICs between two *Salmonella* species at 0.08 µg/mL. WQ-3810 had comparable MICs to ciprofloxacin at 0.04 µg/mL in both *Salmonella* species, which were lower than that of WQ-3034 and WQ-3154.

**TABLE 4:** MICs of the quinolones against *S.* Typhimurium and *S.* Enteritidis

Quinolones	MIC ± SD (μg/mL)^*a*^ ^,^ ^*b*^	
	*S.* Typhimurium	*S.* Enteritidis
WQ-3034	0.08 ± 0.00	0.016 ± 0.00
WQ-3154	0.08 ± 0.00	0.08 ± 0.00
WQ-3810	0.04 ± 0.00	0.04 ± 0.00
Ciprofloxacin	0.04 ± 0.00	0.04 ± 0.00

^*a*^ MIC, minimum inhibitory concentration.

^*b*^ SD, standard deviation.

## DISCUSSION

From the supercoiling assay, WQ-3034 demonstrated the highest, whereas ciprofloxacin exhibited the lowest inhibitory effect against wild-type and mutant *S*. Typhimurium DNA gyrases with amino acid substitution at codon 83 and/or 87. Interestingly, WQ-3034, WQ-3154, and WQ-3810 exhibited a significantly greater inhibitory effect than ciprofloxacin against double mutation of DNA gyrase. These WQ-fluoroquinolones have identical substitutions at the R_1_ position with a heteroaromatic ring called 6-amino-3,5-difluoropyridine-2-yl. This difluoropyridine moiety is structurally larger than cyclopropyl moiety, a functional group of ciprofloxacin. Since the 6-amino-3,5-difluoropyridine-2-yl as a substituent at the R_1_ position of WQ-3810 was proposed to be closer to the amino acid at position 83 and likely to assist WQ-3810 for robust interaction with DNA gyrases (29, 30), WQ-3034 and WQ-3154 may have similarly obtained superior inhibitory activity.

The alteration at the R_7_ position of the quinolone ring has been proposed to increase antibacterial activity (31, 32). A previous study of WQ-3810 revealed that the addition of 3-isopropylaminoazetizine-1-yl at the R_7_ position was inferred to lower IC_50s_ compared to ciprofloxacin and nalidixic acid (29). In the present study, 3-hydroxyazetidinyl as a substituent at the R_7_ position of WQ-3034 and WQ-3154 also resulted in lower IC_50_s compared to ciprofloxacin.

The substitution at the R_8_ position in the quinolone ring was previously proposed to increase the drug activity against anaerobes and gram-positive bacteria (33, 34). Specifically, adding Cl atom and methyl substituents to the R_8_ position was verified to enhance antimicrobial potency against both gram-positive and gram-negative bacteria (35). Here, the R_8_ position of the quinolone ring has the addition of Cl atom in WQ-3034 and methyl group in WQ-3154 and WQ-3810. The IC_50_ against double mutation was 59-fold, 69-fold, 110-fold, and over 7,000-fold higher than WT in WQ-3034, WQ-3154, WQ-3810, and ciprofloxacin, respectively. These enormous differences could be the result of the interaction of molecule at the R_8_ position with mutant gyrases. Similar results were observed in the previous study where the correlation between R_8_ substitution and the lower IC_50_ as well as the interaction of R_8_ substitution of quinolone ring with double-mutant gyrase was proposed (36). Moreover, the addition of the Cl atom at the R_8_ position exerts a robust electron-withdrawing effect on the heteroaromatic ring at R_1_ and stabilizes the molecule (24). By considering the modification of the quinolone ring of all antibacterial compounds in this study, the combination of substituted 6-amino-3,5-difluoropyridine-2-yl at the R_1_ position, 3-hydroxyazetidinyl at the R_7_ position, and addition of Cl atom at the R_8_ position of WQ-3034 may have contributed to the most potent inhibitory effect in WT and mutant *S*. Typhimurium DNA gyrases with amino acid substitution at codon 83 and/or 87.

Among the WQ compounds, WQ-3810 had the lowest MIC, suggesting the influence of the alteration at the R_7_ position. The addition of substituents at the R_7_ position was suggested to facilitate the enhancement of half-life and bacterial tissue penetration (37). Nevertheless, when compared to ciprofloxacin, the MICs of WQ-3034 and WQ-3154 were higher, which could be due to less permeability and accumulation. For the bactericidal effect, the quinolones must be taken up by the bacteria and accumulated in the bacterial cell until reaching the concentration that can inhibit DNA gyrase activity. In this case, WQ-3034 and WQ-3154 may have less permeability and are easier to excrete from the bacterial cell than ciprofloxacin. In addition, the cyclopropyl group at the R_1_ position of ciprofloxacin increases the volume of distribution in the bacterial cell, and manipulation at the R_7_ position extends the half-life of the agent by increasing the lipophilicity (8). Since the previous studies have shown the correlation between IC_50_ and MIC in quinolones (29, 38), the MICs of WQ-3034, WQ-3154, WQ-3810, and ciprofloxacin against mutant DNA gyrases were estimated from IC50s. Assuming the WQ compounds and ciprofloxacin have similar drug efflux transporting system and comparable permeability, when focusing on double amino acid substitution, the MICs of WQ-3034, WQ-3154, WQ-3810, and ciprofloxacin were 4.732, 5.578, 6.635, and 145.278 µg/mL, respectively. Taking into consideration, WQ-3034, WQ-3154, and WQ-3810 will likely be effective quinolone antibacterial drugs due to a higher inhibitory activity than ciprofloxacin and a higher antimicrobial activity against double amino acid substitution strain.

As mentioned previously, MICs against *S*. Typhimurium and *S*. Enteritidis of WQ compounds were higher than ciprofloxacin. This may indicate that WQ compounds were poorly accumulated or easier to excrete from the bacterial cell, which could be the result of low permeability or efflux pumps. In Gram-negative bacteria, the permeability barrier consists of inner and outer membranes (39). The permeability of the compounds across the outer membrane is a crucial factor in drug accumulation in a bacterial cell. Generally, quinolones use both a lipid-mediated and a porin-mediated pathway (40). A previous study demonstrated that membrane permeabilizers such as Tris/ethylenediaminetetraacetic acid (EDTA), polymyxin B, polymyxin B nonapeptide, and guanidinylated polymyxins have the ability to increase the sensitivity of *E. coli* and *S*. Typhimurium to several hydrophobic antibiotics (41, 42). Efflux pumps are proteins on the bacterial cell membrane that the bacteria use for substrate excretion. Since the efflux pump may take part in the WQ compounds extrusion, it could be worthwhile to consider using efflux pump inhibitors in combination with the antimicrobial compounds. The efflux pump inhibitors are the chemical entities that inhibit efflux pumps of the bacteria by one or more mechanisms such as direct binding to the efflux pump to prevent the substrate binding to the active site, subsiding the energy mechanism accountable for the pumps, and chelating iron required for the pumps (43, 44). In light of this, the membrane permeabilizer and efflux pump inhibitors could potentially increase the concentration of WQ compounds inside the bacterial cells. While ciprofloxacin is well studied, the physiochemical properties of WQ-3034 and WQ-3154 are truly limited. Further study is needed to substantially evaluate the efficacy of utilizing the membrane permeabilizer and efflux pump inhibitors in conjunction with WQ compounds to enhance antimicrobial activity and drug effectiveness against pathogenic bacteria.

## MATERIALS AND METHODS

### Antimicrobial compounds and reagents

WQ-3034, WQ-3154, and WQ-3810 were provided by Wakunaga Pharmaceutical Co., Ltd. (Osaka, Japan). Ciprofloxacin was purchased from LKT Laboratories, Inc. (St. Paul, MN, USA). The chemical structures of antimicrobial compounds used in this study, along with the basic structure of quinolones, are presented in Table 1; Fig. 2. Relaxed and supercoiled pBR322 DNA were procured from John Innes Enterprises, Ltd. (Norwich, UK).

### Protein expression and purification

**Fig 7:**
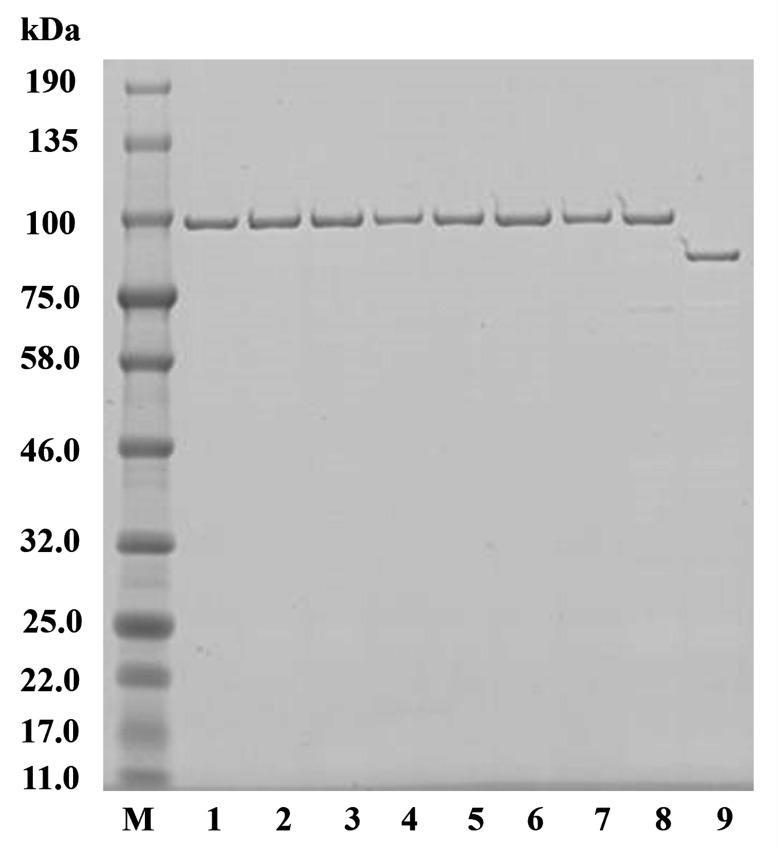
SDS-PAGE (5–20) (ATTO, Tokyo, Japan) of WT and mutant *S.* Typhimurium DNA gyrase subunits. Approximately 300 ng of each protein subunit was loaded into each well. Lane M: protein size markers (kDa; Bio-Rad Lab. Inc., Japan); lane 1: WT *S.* Typhimurium GyrA subunit; lanes 2–8: Ser83Ile, Ser83Phe, Ser83Tyr, Asp87Gly, Asp87Asn, Asp87Tyr, and Ser87Phe-Asp87Asn *S.* Typhimurium GyrA mutants, respectively; and lane 9: WT *S.* Typhimurium GyrB subunit.

Recombinant DNA gyrase was obtained as separate subunits, GyrA and GyrB. In total, plasmids encoding nine subunits including WT GyrA and GyrB as well as seven GyrA with amino acid substitutions (Ser83Phe, Ser83Ile, Ser83Tyr, Asp87Gly, Asp87Asn, Asp87Tyr, and Ser87Phe-Asp87Asn) in the quinolone resistance determining region were constructed as described in our previous study (38). These plasmids were introduced into *Escherichia coli* BL21(DE3) (Merck KGaA, Darmstadt, Germany), and protein expression was induced in the bacterial cells. A transformed *E. coli* was inoculated into Luria-Bertani broth supplemented with ampicillin (50 mg/L) and incubated at 37°C until the optical density value reached 0.60. Expression of GyrA and GyrB was induced by adding 1 mM isopropyl beta-d-1-thiogalactopyranoside (Wako Pure Chemical Industries Ltd, Osaka, Japan) followed by incubation at 16°C for 40 h (for GyrA), or 18°C for 13 h (for GyrB). To release the expressed protein, *E. coli* cells were harvested by centrifugation, suspended in a native binding buffer containing complete mini EDTA-free (Roche Applied Science, Mannheim, Germany), and then sonicated at a 30% duty cycle with 10 cycles of 40 s on and 40 s off (Sonifier 250; Branson, Danbury, CT). The recombinant DNA gyrase subunits were purified by column chromatography using Ni-NTA agarose resin (Thermo Fisher Scientific, Inc., Waltham, MA) and dialyzed against DNA gyrase dilution buffer [50 mM Tris-HCl, pH 7.5; 100 mM KCl, 2 mM dithiothreitol (DTT), 1 mM EDTA]. The obtained protein was mixed with glycerol to prevent denaturation and stored in small aliquots at −80°C for future use. The protein purity was assessed by sodium dodecyl sulfate-polyacrylamide gel electrophoresis (SDS-PAGE) (ATTO, Tokyo, Japan) as shown in Fig. 7.

### Fluoroquinolone-inhibited DNA supercoiling assay

Fluoroquinolone-inhibited DNA supercoiling assay was performed to assess the inhibitory effects of WQ-3034, WQ-3154, WQ-3810, and ciprofloxacin. The 30 µL of reaction mixture contained DNA gyrase reaction buffer [35 mM Tris-HCl, 6 mM MgCl_2_, 1.8 mM spermidine, 24 mM KCl, 5 mM DTT, 0.36 mg/mL of BSA, and 6.5% glycerol (wt/vol)], 50 mM ATP, 18 nM GyrA, 18 nM GyrB, 1.5 nM relaxed pBR322 DNA, and serially diluted fluoroquinolones. WQ-3034, WQ-3154, and WQ-3810 were used at the concentration of 0.01–5.12 µg/mL (for WT, Ser83Phe, Ser83Tyr, Asp87Gly, Asp87Asn, and Asp87Tyr) and 0.04–20.48 µg/mL (for Ser83Ile and Ser87Phe-Asp87Asn). While ciprofloxacin was used at four different concentration ranges: 0.01–5.12 µg/mL (for WT, Ser83Phe, Asp87Gly, and Asp87Tyr); 0.04–20.48 µg/mL (for Ser83Tyr and Asp87Asn); 0.08–40.96 µg/mL (for Ser83Ile); and 3.20–1,638.4 µg/mL (for Ser87Phe-Asp87Asn). The reaction mixture was incubated at 37°C for 60 min and then the reaction was halted by adding 8 µL of stop solution (5% SDS, 25% glycerol, and 0.25 mg/mL of bromophenol blue) (45). Next, 10 µL of each reaction mixture was subjected to gel electrophoresis for 80 min at 50 mA in 1.2% agarose gel in 1×Tris-acetate-EDTA (TAE) buffer. Subsequently, the gel was stained with 0.5 µg/mL of ethidium bromide for 30 min. The presence of supercoiled DNA can be easily distinguished as a separated band under the UV light. The amount of supercoiled DNA was quantified by its intensity using ImageJ, the digital image processing software (https://imagej.nih.gov/ij/download.html). The concentration of the compounds that reduce 50% DNA gyrase activity (IC_50_) was calculated with the AAT Bioquest IC_50_ Calculator web tool (https://www.aatbio.com/tools/ic50-calculator).

### Fluoroquinolone-mediated DNA cleavage assay

DNA cleavage assays against WT and Ser83Ile were conducted as previously described (46, 47). Instead of relaxed form, supercoiled pBR322 DNA was utilized as the substrate for the cleavage assays. The 30-µL reaction mixture consisted of DNA gyrase assay buffer, purified DNA gyrase subunits, supercoiled pBR322 DNA (0.3 µg), and increasing concentrations of WQ-3034 and ciprofloxacin. WQ-3034 was used at the concentration of 0.01–5.12 µg/mL for WT and at 0.01–100 µg/mL for Ser83Ile. Ciprofloxacin was used at the concentration of 0.01–33.33 µg/mL for both of DNA gyrases. Following an incubation period of 1 h at 37°C, 3 µL of 2% SDS and 3 µL of proteinase K (1 mg/mL) were added to the reaction mixture. After an additional 30 min incubation at 37°C, the reactions were stopped by adding 8 µL of stop solution. Subsequently, 10 µL of each reaction mixture was subjected to gel electrophoresis for 96 min at 50 mA in 1% agarose gel in 1×TAE buffer and stained with 0.5 µg/mL of GelRed (Biotium, Hayward, CA, USA) for 30 min. Plasmid pBR322 linearized by BamHI digestion was used as cleaved DNA marker. The gel was photographed under the UV illumination, and the extent of DNA cleavage was quantified by ImageJ (https://imagej.nih.gov/ij/download.html). The quinolone concentrations that require to induce 25% of maximum DNA cleavage (CC_25_s) were determined for WQ-3034 and ciprofloxacin.

### Antimicrobial susceptibility testing for nontyphoidal *Salmonella*

The antimicrobial activity of WQ-3034, WQ-3154, WQ-3810, and ciprofloxacin was tested against *S*. Typhimurium NBRC 13245 and *S*. Enteritidis NBRC 3313 using micro-broth dilution method as a recommended protocol of the Clinical and Laboratory Standards Institute (48). A suspension containing approximately 5 × 10^5^ CFU/mL of each *Salmonella* strain was transferred into 96-well plate containing serially diluted fluoroquinolones and incubated at 37°C for 16 h. The minimum inhibitory concentration was defined as the lowest FQs concentration required to entirely inhibit the visible bacterial growth in the well.
